# Microstructure and High-Temperature Ablation Behaviour of Hafnium-Doped Tungsten-Yttrium Alloys

**DOI:** 10.3390/ma16062529

**Published:** 2023-03-22

**Authors:** Rui Wu, Chuanbing Huang, Huifeng Zhang, Haozhong Lv, Xiaoming Sun, Hao Lan, Weigang Zhang

**Affiliations:** 1 School of Rare Earths, University of Science and Technology of China, Hefei 230026, China; 2 Ganjiang Innovation Academy, Chinese Academy of Sciences, Ganzhou 341119, China; 3 Institute of Process Engineering, Chinese Academy of Sciences, Beijing 100190, China; 4 School of Chemical Engineering, University of Chinese Academy of Sciences, Beijing 100049, China

**Keywords:** tungsten-yttrium-hafnium alloy, HfW_2_, ablation resistance, HfO_2_ oxide layer

## Abstract

W is a widely used refractory metal with ultra-high melting point up to 3410 °C. However, its applications are limited by poor ablation resistance under high-temperature flame and air flow, which is crucial for aerospace vehicles. To improve the ablation resistance of W under extreme conditions, W-Y alloys doped with different Hf mass fractions (0, 10, 20, and 30) were prepared using the fast hot pressing sintering method. Microstructure and ablation behaviours at 2000 °C were investigated. Results showed that adding an appropriate amount of Hf improved the properties of the W-Y alloy evidently. In particular, the hardness of the alloy increased with the increased content of Hf. The formation of the HfO_2_ layer on the surface during ablation decreased the mass and linear ablation rates, indicating enhanced ablation resistance. However, excessive Hf addition will result in crack behaviour during ablation. With a Hf content of 20 wt.%, the alloy exhibited high stability and an excellent ablation resistance.

## 1. Introduction

W is a refractory metal with the highest melting point of 3410 °C compared to those of the others. In addition, it exhibits excellent properties, such as high density, high chemical stability, low vapor pressure, and low coefficients of thermal expansion, which increase its wide applicability [1,2,3,4,5]. Recently, the high-temperature applications of W, such as in fusion reactors and hypersonic vehicles, have received considerable attention. In particular, the first wall materials, missile nose caps, and rocket nozzles are representative components operating in harsh external environments, such as high temperature of over 2000 °C and strong airflow scouring [6]. However, the application of W in high-temperature scenarios is limited by its poor oxidation and ablation resistance. In detail, W starts to oxide at 300–400 °C and forms WO_x_ (x = 2~3) at higher temperatures, exhibiting a low melting point of 1473 °C, resulting in a poor resistance of the oxide layer. Therefore, the oxidation and ablation resistance of W should be improved to withstand higher service temperatures [7].

In order to improve this situation, researchers hope to improve the ablation resistance of W by the alloying method. Now, several studies have confirmed the benefits of the addition of metals [8,9,10,11,12]. However, these metals cannot withstand high-temperature ablation, and their oxides exhibit poor stability, similar to W. Therefore, how to improve the ablation resistance should be further investigated. Hf is also a refractory metal with a high melting point of 2233 °C. Higher melting point oxide (HfO_2_) will be formed at high temperatures. Based on W-Hf phase diagram [13], HfW_2_ formed by diffusion during sintering also has a high melting point and microhardness, implying excellent properties of the W-Hf alloy. However, as Hf is located in the IVB region of the periodic table, similar to Zr, its high activity results in the oxidation after sintering [14,15]. To address this issue, rare earth (RE) elements, which have high affinity toward O and impurities, can be used to prevent the oxidation of Hf. RE-doped W alloys possess notable characteristics, such as high-temperature strength and wear properties, and they demonstrate high performance in purifying the matrix and pinning the grain boundary [16,17,18,19].

Y is more active than Hf, and its corresponding oxide (Y_2_O_3_) is highly stable. Previous studies have employed Y and Y_2_O_3_ to promote sintering [20]. As one of rare earth oxides, Y_2_O_3_ is an excellent sintering aid with a low W eutectic point of 1560 °C [21]. Therefore, it is an optimal reinforcement for oxide-dispersion-strengthened W [22,23,24,25]. Though Y will be oxidized for the absorption to oxygen, the formed Y_2_O_3_ will not have a negative effect on the matrix. Further, the addition of RE elements in their metallic state is more effective than that of their oxidic state [26].

Owing to the poor sintering performance, the fabrication of fully dense W by hot pressing requires a temperature more than 2200 °C, and it will prompt grain growth [25]. To address this issue, fast hot pressing (FHP) sintering is proposed as a new technology to complete the sintering process with the assistance of current within minutes, thereby saving the sintering time and inhibiting grain growth.

In this study, W-1.5 wt.% Y-x wt.% Hf (x = 0, 10, 20, 30) alloys (shortened as W-1.5 Y-x Hf) were fabricated using the FHP method to investigate their microstructure and ablation behaviour based on a previous study [27]. This study evaluated the effects of Hf on W-Y alloys during high-temperature ablation and elucidated the ablation mechanisms with Hf addition.

## 2. Materials and Methods

Pure W (1–3 μm, 99.9% purity), YH_2_ (~74 μm, 99% purity), and Hf (~10 μm, 99.9% purity) powders were prepared for the experiment. Different mass fractions of Hf (0, 10, 20, and 30) were added to W-1.5 wt.% Y alloy powders used as the starting materials. The powders were mixed in a planetary ball mill (ZQM-4L, Tianchuang, Changsha, China) using zirconia balls with a ball-to-powder ratio of 5:1 and speed of 280 rpm for 8 h. Ethanol was used as the medium. The ball-milled powders were filtered and vacuum dried for sintering (FHP-828, Hateng, Suzhou, China). The powder was placed in a graphite mould and sintered at 1800 °C for 3 min under an Ar atmosphere at a pressure of 50 MPa. The heating rate was maintained at 100 °C/min up to 1600 °C and 50 °C/min up to 1800 °C.

The diameter of sintered specimens is 30 mm, and the height is more than 3 mm (to optimize the process for ablation). The mass contents of powders are calculated and weighed to prepare the composition of sintered specimen. For example, for 100 g W-1.5 Y-10 Hf powder, the mass of different elements can be calculated as follows:

$$m=m_{W}+m_{Y}+m_{Hf}=100\;g$$

$$m_{Y}=100\times 1.5\%$$

$$m_{Hf}=100\times 10\%$$

$$m_{W}=100-m_{Y}-m_{Hf}$$

The sintered specimens were ground to 2000 mesh and polished using the diamond polishing agent. The phase composition was analysed via X-ray diffraction (XRD, X pert Pro MPD, PANalytical B.V., Almelo, NL, USA). Field emission scanning electron microscopy (FE-SEM) coupled with energy-dispersive spectroscopy (JSM-7001 F (JEOL, Akishima, Tokyo) + INCA X-MAX (Oxford Instruments, Abbington, Oxford, UK)) was employed to investigate the microstructure and elemental distribution. A transmission electron microscopy (TEM) instrument integrated with an EDS system (FEI Talos F200x G2 + super-x, Thermo Fisher Scientific, Waltham, MA, USA) was used to investigate the crystal structure.

Vickers microhardness (HX-1000 TM, Taiming, Shanghai, China) was tested under a load of 200 g for 15 s. The calculation formula is listed as follows:

$${HV}_{0.2}=\frac{0.1891\times F}{d^{2}}\;(F:\;load;\;d:\;indentation\;diameter)$$

The relative density was measured by the Archimedes method using an electronic balance (ML204/02, Mettler Toledo, Columbus, OH, USA). The calculation progress is concluded as follows:

$$\rho_{relative}=\frac{\rho_{real}}{\rho_{theory}}$$

$$\rho_{real}=\frac{M\times \rho_{0}}{M_{1}}\;(\rho_{0}:\;density\;of\;water;M_{1}:\;mass\;in\;the\;water)$$

$$\rho_{theory}=\frac{M}{{\Sigma V}_{i}}$$

$$V_{i}=\frac{M_{i}}{\rho_{i}}$$

The weight and height of the specimens before and after ablation were recorded to calculate the mass and linear ablation rates. The ablation test was performed in an Ar-H plasma ablation system that can provide high temperatures of more than 2000 °C. The distance between spray nozzle and surface of specimen was set to 45 mm. During temperature detection using an infrared thermometer (RAYMM1MHVF1L, Raytek, Santa Cruz, CA, USA), the ablation condition was controlled at 2000 °C for 60 s. The surface and cross-section of the ablated specimen were also investigated.

## 3. Results and Discussion

### 3.1. Microstructure Analysis

#### 3.1.1. Composition Analysis

**Figure 1:**
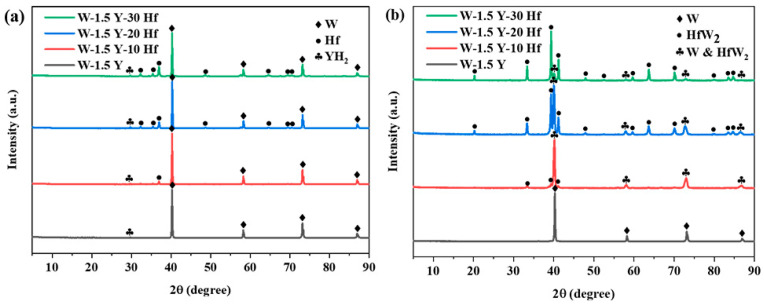
XRD patterns of the ball-milled powders (**a**) and sintered specimens (**b**).

The XRD results of the milled powders and sintered specimens are shown in Figure 1. The peaks of milled powders in Figure 1a are identified as W, Hf, and YH_2_, respectively, corresponding to the starting materials. Figure 1b shows the results of the sintered specimens. Four characteristic peaks identified as W were observed on each curve, whereas Y peaks were not noted for relatively weak peak intensity. With the increase in Hf content, different peaks corresponding to HfW_2_ appeared with increasing intensity. Besides, some peaks of HfW_2_ phase are highly coincident with W [13], leading to the combination of two diffraction peaks. The combined peaks are marked using “W & HfW_2_” in order to distinguish them from the others. The phase equilibrium values and SemiQuant values of W and HfW_2_ phases formed after sintering with different contents of Hf are listed in Table 1. The values of SemiQuant are close to phase equilibrium values. According to Table 1, the main phase of matrix will change from W to HfW_2_ when the Hf content reaches 20 wt.%.

**Figure 2:**
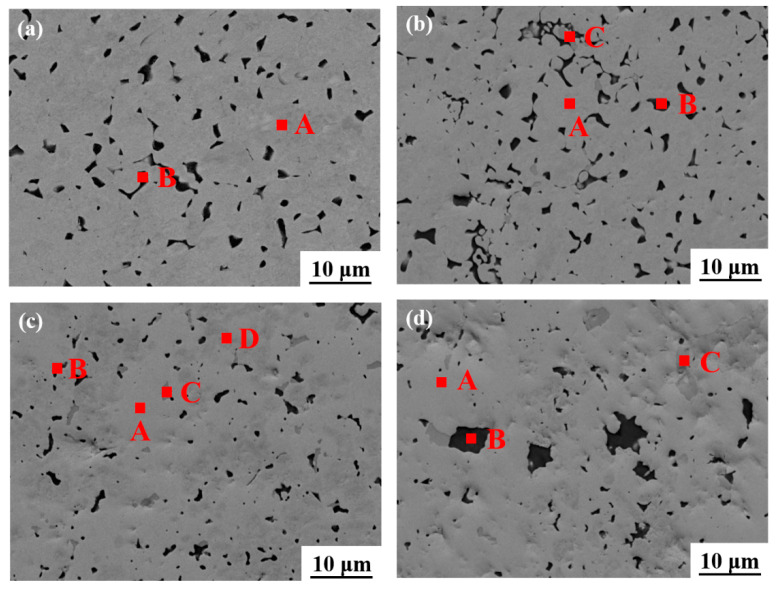
SEM images of the sintered W-1.5 Y-x Hf, where x is (**a**) 0, (**b**) 10, (**c**) 20, and (**d**) 30.

SEM was used to investigate the microstructure of the W-1.5 Y alloys with different Hf contents, as shown in Figure 2a–d. When the Hf content was less than 10%, the microstructure of the samples was retained. When the Hf content was increased to 20 wt.%, the microstructure changes obviously, which is consistent with the above analysis. When the content of Hf reaches 30%, the matrix changes completely, and the black phase agglomerates.

EDS analysis was performed to identify the different phases of the specimen, as listed in Table 2. Two phases of A and B in Figure 2a are, respectively, identified as W and Y_2_O_3_ [26,28]. With 10 wt.% Hf, three phases marked as A, B, and C are noted in Figure 2b. The bright grey phase A is W with a small amount of Hf diffusion. Meanwhile, phase B is still Y_2_O_3_, and the dark grey phase C is Hf dissolved with W. It demonstrates the diffusion of W and Hf within each other at high temperatures. When the Hf content is 20 wt.%, the content of HfW_2_ (A) exceeds that of W (D). Meanwhile, part of W phase still exists in the matrix. However, when the Hf content reaches 30 wt.%, the matrix is basically transformed into HfW_2_, as shown in Figure 2d, which conforms to the results in Table 1.

#### 3.1.2. Relative Density and Microhardness Analyses

The relative density and microhardness values are listed in Table 3. The W-1.5 Y alloy has a lower relative density due to the difficulty of sintering [22,23]. Adding appropriate amounts of Hf improved the density of the alloys. However, the relative density decreased to 96% when the Hf content reached 30 wt.%, indicating that excessive Hf addition inhibited sintering. Nevertheless, the relative density of all specimens exceeds 95%, suggesting the successful sintering and densification of the W-Y-Hf alloys. Meanwhile, the hardness sharply increased up to 1213 HV_0.2_ when the Hf content reaches 30 wt.%. This can be attributed to the improved density, solid solution strengthening effects, and the formation of a large amount of HfW_2_ phase, which has high hardness [13]. Hence, appropriate Hf addition promotes the matrix performance of W alloys.

#### 3.1.3. Structure Analysis

**Figure 3:**
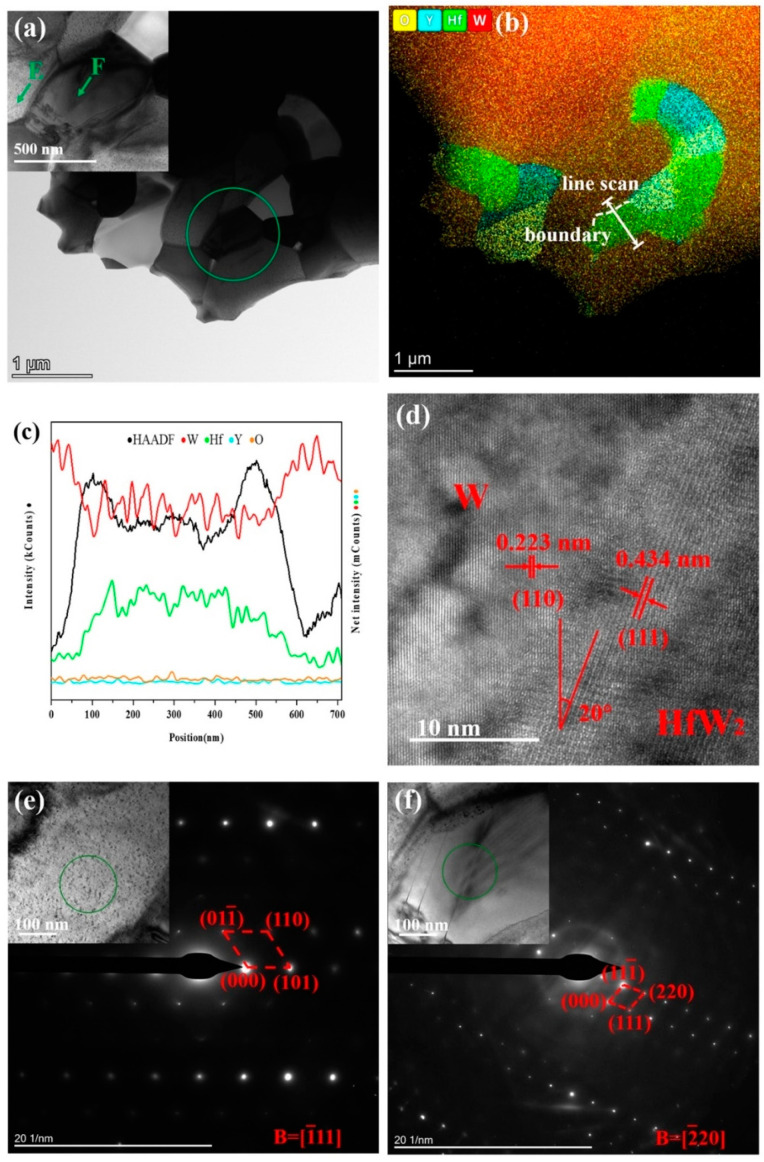
TEM images of the W-1.5 Y-20 Hf alloy. (**a**) Morphology after ion thinning. Inset shows the magnified morphology of the area in the green circle. (**b**) EDS analysis of the elemental distribution of (**a**). (**c**) Line scan in (**b**). The black curve represents the relative intensity of the atom number within the range. (**d**) TEM images at the interface of (**b**). (**e**) Diffraction pattern of E in (**a**). (**f**) Diffraction pattern of F in (**a**).

TEM analysis was used to investigate the microstructure, element distribution, and diffraction patterns of different phases. Figure 3a,b show the morphology of W-1.5 Y-20 Hf after ion-beam thinning and the corresponding element distribution, respectively. The W-rich and Hf-rich phases are obviously different in morphology. O is mainly distributed in the Y-containing area because Y absorbs O to form Y_2_O_3_ and purify the matrix [29]. Figure 3c shows the line scan image of relative intensity of the different elements marked in Figure 3b. As the line scan did not pass through the Y-rich area, Y and O intensity curves are horizontal. W and Hf exhibit diametrically opposite trends when the line scan passed through the Hf-rich area. Meanwhile, the trend of the black curve shows that there are still large amounts of W remaining in Hf, implying the formation of HfW_2_. Figure 3d shows the lattice stripes on both sides of the boundary marked in Figure 3b. The stripe spacing in the left and right areas are 0.223 and 0.434 nm, respectively, which is consistent with the crystallographic W (110) plane and HfW_2_ (111) plane [30]. The included angle between two phases is approximately 20°. Figure 3e,f show the fast Fourier transform results of the circled area in the embedded HR-TEM images. The calculation results correspond to the crystallographic structure of W and HfW_2_. Therefore, adding Hf to the W-1.5 Y alloy resulted in the formation of HfW_2_, confirming the results in Figure 1b.

### 3.2. Ablation Analysis

#### 3.2.1. Ablation Surface Analysis

**Figure 4:**
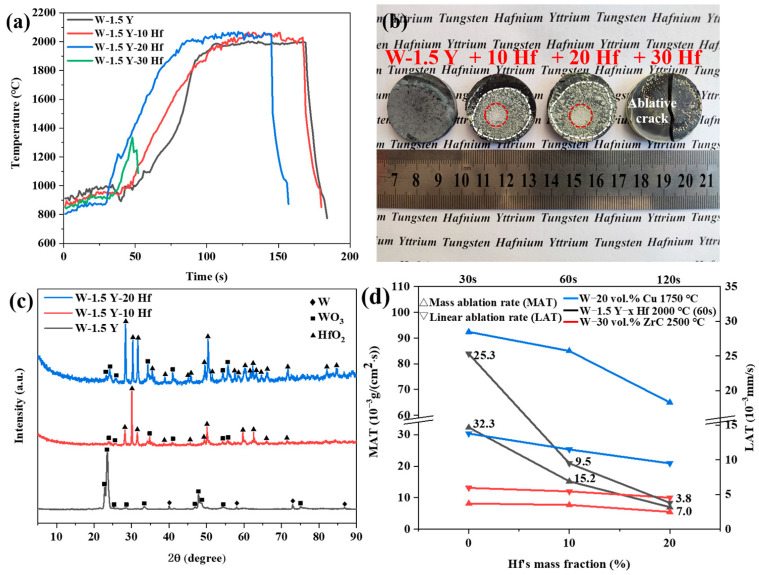
Analysis of the ablated surface. (**a**) Temperature versus time ablation curve. (**b**) Macromorphology of the ablated specimens. Central and surrounding areas are indicated by red and white circles, respectively. (**c**) XRD patterns of the ablated surface and (**d**) mass ablation rate and linear ablation rate.

Figure 4a shows the ablation curve plotted with temperature as a function of time. When the content of added Hf was less than 20 wt.%, the specimens remained stable under temperature variations, thereby withstanding ablation at 2000 °C for 60 s. However, when the Hf content reached 30 wt.%, the specimen cracked at the heating stage. The ablated specimens are shown in Figure 4b. The sizes of alloys remained the same after ablation. As the poor oxidation resistance of W and low melting point of its oxide decrease the ablation resistance, W-1.5 Y has a smooth pit surface without an obvious melting layer. Compared with W-1.5 Y, the alloy added with 10 or 20 wt.% Hf exhibited a flat surface covered with a melting layer. The melting layer of W-1.5 Y-20 Hf was more uniform and denser. Although W-1.5 Y-30 Hf has a flat surface, it is too unstable at high temperatures to withstand ablation. This can be ascribed to the increased press in sintering with the excessive addition of Hf, resulting in cracking behaviour during the high-temperature ablation process [31,32].

Figure 4c shows the surface XRD of ablated specimens. Compared with W-1.5 Y, the ablated surfaces with 10 and 20 wt.% Hf generated large amounts of HfO_2_ phase, implying the formation of Hf oxide layer [33]. The number and intensity of diffraction peaks of ablated W-1.5 Y-20 Hf alloy increased, indicating better protection effects. Figure 4d shows the mass and linear ablation rates of ablated specimens. The mass and linear ablation rates decreased from 32.3 × 10^−3^ to 7.0 × 10^−3^ g/(cm^2^·s) and 25.3 × 10^−3^ to 3.8 × 10^−3^ mm/s, respectively, as the Hf content was increased from 0 to 20 wt.%. Compared with traditional W-Cu alloy, Hf-added alloys have lower mass and linear ablation rate, and they are close to composites added with carbides [34]. These results suggest the significant effect of adding Hf for improving ablation resistance. Based on the results, W-1.5 Y-20 Hf exhibited the highest ablation resistance in this study.

**Figure 5:**
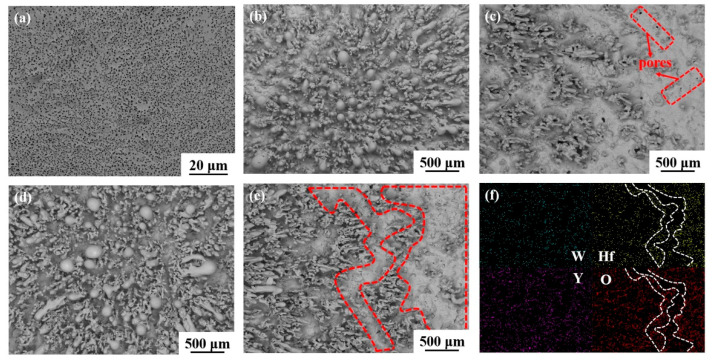
SEM images of the ablated surface. (**a**) Microstructure of W-1.5 Y. (**b**) Central area of ablated W-1.5 Y-10 Hf. (**c**) Surrounding area of ablated W-1.5 Y-10 Hf. (**d**) Central area of ablated W-1.5 Y-20 Hf. (**e**) Surrounding area of ablated W-1.5 Y-20 Hf. (**f**) EDS analysis of the element distribution in (**e**).

The topographies of ablated surfaces of W-1.5Y alloys added with different Hf contents are shown in Figure 5. Figure 5a shows the smooth ablated surface of W-1.5 Y alloy, which is consistent with the results in Figure 4b. However, many pores are distributed on the surface layer, which may be attributed to the porous WO_3_ and dissolution of Y_2_O_3_ in the WO_3_ melt during the high-temperature ablation process [35,36]. The low melting point of WO_3_ and porous structure lead to the poor performance of surface layer of W-1.5 Y in resisting oxidation and high-temperature ablation. Figure 5b,c show the central and surrounding areas of the ablated surface of W-1.5 Y-10 Hf. After cooling, many melted oxides were generated, attached, and aggregated in the central area, whereas some were blown to the surrounding area. In contrast to W-1.5 Y, Hf addition resulted in the generation of HfO_2_ during ablation. As HfO_2_ has a good ablation resistance owing to its high melting point of ~2800 °C, a melted oxide layer covering the alloy surface was observed, though some pores can be found. Figure 5d,e show the increased content of the melted oxides and compactness of the surface layer, as indicated by the red-circled area when the Hf content reached 20 wt.%. EDS analysis was used to investigate the elemental distribution and compositions of the surface layer, as shown in Figure 5f. W and Y were evenly distributed in the scanning area. In contrast, Hf was concentrated in the right flat area, as marked by the white line. The O distribution coincides with that of Hf, indicating the formation of a HfO_2_ protective layer.

#### 3.2.2. Ablation Cross-Section Analysis

**Figure 6:**
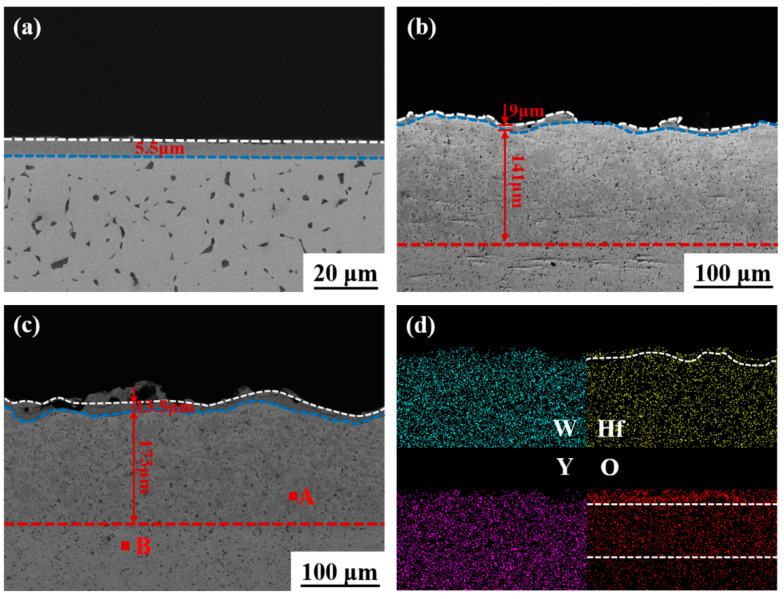
Ablation analysis of the sample cross-section: (**a**) W-1.5 Y, (**b**) W-1.5 Y-10 Hf, and (**c**) W-1.5 Y-20 Hf. (**d**) EDS analysis of the element distribution in (**c**).

Figure 6a shows the cross-section of ablated W-1.5 Y, whereby an oxide layer covering the surface can be clearly observed. The average thickness of the oxide layer is approximately 5.5 µm. The flat local oxide layer corresponds well with the smooth surface in Figure 5a. Figure 6b,c show the ablated cross-section of alloys with 10 and 20 wt.% Hf. The alloy structure can be divided into three parts, namely, the melted oxide layer, the oxygen diffusion area, and the internal matrix. The thicknesses of the oxide layer and oxygen diffusion area are 9 and 141 µm, respectively, in Figure 6b, as well as 13.5 and 173 µm, respectively, in Figure 6c. The existence of oxygen diffusion area demonstrates that Hf addition has considerable effects on protecting the internal matrix by forming a surface layer. Compared to W-1.5 Y-10 Hf, W-1.5 Y-20 Hf has a thicker oxide layer with higher uniformity and coverage, which is beneficial in preventing the infiltration of oxygen and reducing the WO_3_ loss [33,37]. Therefore, W-1.5 Y-20 Hf has a higher ablation resistance, indicating a more protective effect on the matrix. This also explains the larger oxygen diffusion depth in W-1.5 Y-20 Hf than that of W-1.5 Y-10 Hf [6]. Two phases in the oxygen diffusion area caused by oxidation are marked in Figure 6c. The element analysis results are listed in Table 4. The bright grey phase is the primitive HfW_2_ phase, and the dark grey phase is the W-Hf-O compound. Figure 6d shows the EDS analysis of the cross-section of Figure 6c. W and Y are uniformly distributed in the scanning area. The top distribution outline of Hf marked in white line matches well with the surface profile, demonstrating the formation of the HfO_2_ layer. Moreover, O distribution regions can be divided into three types: the upper region represents the melted oxide layer, the middle region represents the partially oxidised W-Hf compound, and the lower region contains only a small amount of oxygen.

#### 3.2.3. Ablation Mechanism

**Figure 7:**
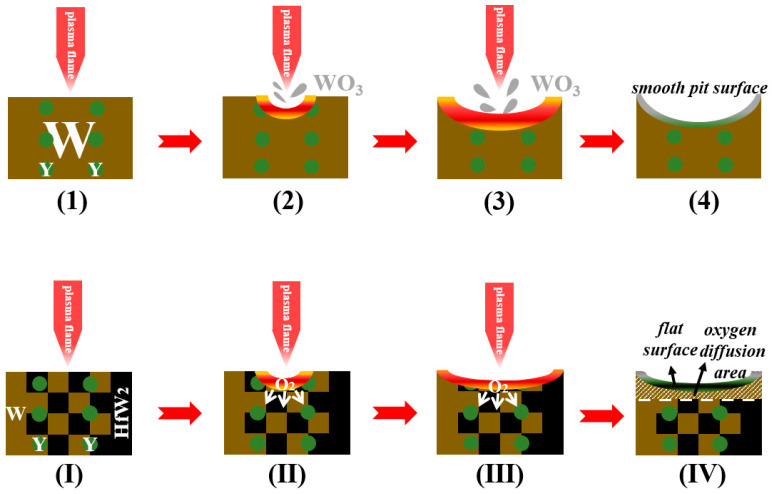
Ablation mechanism of (**1**–**4**) W-1.5 Y and (**I**–**IV**) W-1.5 Y-x Hf.

Among the sintered samples, Hf-added W-1.5 Y alloys exhibit better properties. Figure 7 depicts the ablation process and ablation mechanism of the W-Y and W-Y-Hf alloys. For W-1.5 Y, the surface was instantaneously oxidised to WO_3_ when the temperature reached 2000 °C. Due to its low melting point, WO_3_ formed on the surface became liquid and was blown away by the plasma plume. The main reaction is:

2W + 3O_2_ → 2WO_3_ (1)

Due to the significant loss of W, the specimen exhibited a smooth surface after cooling. Consequently, the mass and height significantly decreased, particularly near the centre, thereby forming a crater-like surface. Therefore, the high-temperature applications of W-1.5 Y are severely limited.

The ablation mechanism changed when Hf was added as an anti-ablation component to protect the matrix. Under the same ablation condition, oxidation occurred, thereby forming WO_3_ and HfO_2_. During the high-temperature ablation process, the W gradually lost as WO_3_ was melted, whereas HfO_2_ remained in the matrix, forming the composite oxide layer. The altered reactions can be summarised as follows:

2W + Hf → HfW_2_ (2)

2W + 3O_2_ → 2WO_3_ (3)

HfW_2_ + 4O_2_ → 2WO_3_ + HfO_2_ (4)

HfW_2_ + XO_2_ → HfW_2_O_2X_ (5)

The formation of HfO_2_ layer with a high melting point improved the ablation resistance by decelerating the oxygen diffusion and reducing the W loss. Therefore, the W-Y-Hf alloys exhibit flat surfaces with lower mass and linear ablation rates.

## 4. Conclusions

This study is mainly focused on investigating the microstructure and ablation behaviour of W-1.5 Y-x Hf (x = 0, 10, 20 30) alloys. The results obtained in this study can be summarised as follows:

(1) The prepared powders sintered using the FHP method achieved high relative densities of more than 95%. Hf was retained after sintering with the protection effect of Y. W and Hf formed the HfW_2_ phase via diffusion. The microhardness increased from 501 to 1213 HV_0.2_ with the increase in Hf content from 0 to 30 wt.%.
(2) After ablation, the W-1.5 Y alloy had a smooth pit surface, whereas W-Y-Hf alloys had a flat surface covered with an obvious oxide layer. When the Hf content was increased from 0 to 20 wt.%, the mass and linear ablation rates decreased from 32.3 × 10^−3^ to 7.0 × 10^−3^ g/(cm^2^·s) and 25.3 × 10^−3^ to 3.8 × 10^−3^ mm/s, respectively.
(3) Under the same ablation conditions, the Hf-added W-1.5 Y alloys form a more protective composite oxide layer. This slowed the diffusion of oxygen and reduced the ablation loss, thereby improving the ablation resistance and protecting the matrix. In view of the result, adding Hf will be of benefit to improve the ablation resistance of W. W-Y-Hf alloys can be developed to fabricate the high-temperature matrix in the aerospace field or for components and parts for high-temperature ablation. However, adding too much Hf results in an ablative crack during the high-temperature ablation process. A comprehensive analysis revealed the W-1.5 Y alloy added with 20 wt.% Hf as the optimal composition.

## Figures and Tables

**Table 1:** Phase equilibrium values and SemiQuant values of different phases in alloys.

Proportion (wt.%)	W		HfW_2_	
	Phase Equilibrium Value	SemiQuant Values	Phase Equilibrium Value	SemiQuant Values
W-1.5Y-10Hf	80	75	20	25
W-1.5Y-20Hf	45	49	55	51
W-1.5Y-30Hf	11	6	89	94

**Table 2:** EDS analysis results revealing the element content at different points in Figure 2.

Compositions (at.%)	W	Hf	Y	O
(a) _A_	100.00	—	—	—
(a) _B_	6.57	—	29.66	63.77
(b) _A_	93.13	6.87	—	—
(b) _B_	—	—	34.78	65.22
(b) _C_	12.08	87.92	—	—
(c) _A_	65.82	34.18	—	—
(c) _B_	—	4.32	29.48	66.20
(c) _C_	—	87.66	—	12.34
(c) _D_	100.00	—	—	—
(d) _A_	64.43	35.57	—	—
(d) _B_	—	2.33	41.25	56.42
(d) _C_	—	100.00	—	—

**Table 3:** Relative density and hardness of the W-1.5 Y alloys with the addition of different Hf contents.

	Relative Density	Hardness (HV_0.2_)
W-1.5 Y	96.3%	501 ± 15
W-1.5 Y-10 Hf	99.8%	655 ± 13
W-1.5 Y-20 Hf	98.9%	950 ± 13
W-1.5 Y-30 Hf	96.0%	1213 ± 16

**Table 4:** Element analysis of the points in Figure 6c.

Composition (at.%)	W	Hf	O
A	35.02	17.84	47.14
B	66.90	33.10	0.00

## Data Availability

Not applicable.
